# Agro-industrial by-products valorization for fructooligosaccharide production with *Zymomonas mobilis*

**DOI:** 10.1186/s40643-025-00887-4

**Published:** 2025-10-07

**Authors:** Adelaide Braga, Ana Benedita Maia, Lígia R. Rodrigues

**Affiliations:** 1 CEB—Centre of Biological Engineering, Universidade Do Minho, Campus de Gualtar, 4710-057 Braga, Portugal; 2 LABBELS—Associate Laboratory, Braga, Guimarães, Portugal

**Keywords:** Agro-industrial by-product, Corn steep liquor (CSL), Fructooligosaccharides (FOS), Molasses, One-step bioprocess, *Zymomonas mobilis*

## Abstract

Fructooligosaccharides (FOS) have gained attention due to their prebiotic properties and potential health benefits. This study explores the production of FOS using *Zymomonas mobilis* ZM4, a promising candidate for biotechnological processes, utilizing corn steep liquor (CSL) and sugarcane molasses as alternative and sustainable carbon and nitrogen sources. Two distinct media formulations were investigated, namely one composed of CSL supplemented with yeast extract (YE), and another utilizing sugarcane molasses. CSL was evaluated at concentrations of 10 g L^−1^ and 12 g L^−1^ in combination with YE. The optimal combination, 12 g L^−1^ CSL and 8 g L^−1^ YE, yielded 60.00 ± 0.44 g L^−1^ FOS with a productivity of 1.250 ± 0.009 g L^−1^ h^−1^, comparable to synthetic media. Molasses, another agro-industrial by-product, was tested at sucrose-equivalent concentrations of 150, 200, and 350 g L^−1^. The highest FOS concentration, 58.67 ± 1.64 g L^−1^, was achieved with 200 g L^−1^ of molasses. Combining CSL and molasses (CSLM media) resulted in 58.15 ± 0.21 g L^−1^ of FOS with a yield of 0.307 ± 0.003 g_FOS_ g_sucrose_^−1^. The FOS mixture included 1-kestose, 6-kestose, nystose, and neokestose. Although scaling up to a bioreactor led to a lower FOS concentration of 42.31 ± 0.16 g L^−1^, the yield remained promising at 0.482 ± 0.008 g_FOS_ g_sucrose_^−1^. This study not only highlights the efficient production of FOS using *Z. mobilis* ZM4 but also demonstrates the potential of using CSL and molasses, byproducts of agro-industrial processes, as cost-effective and sustainable substrates for industrial-scale FOS production. The findings provide valuable insights for the development of bio-based processes for functional oligosaccharide production.

**Graphical abstract:**

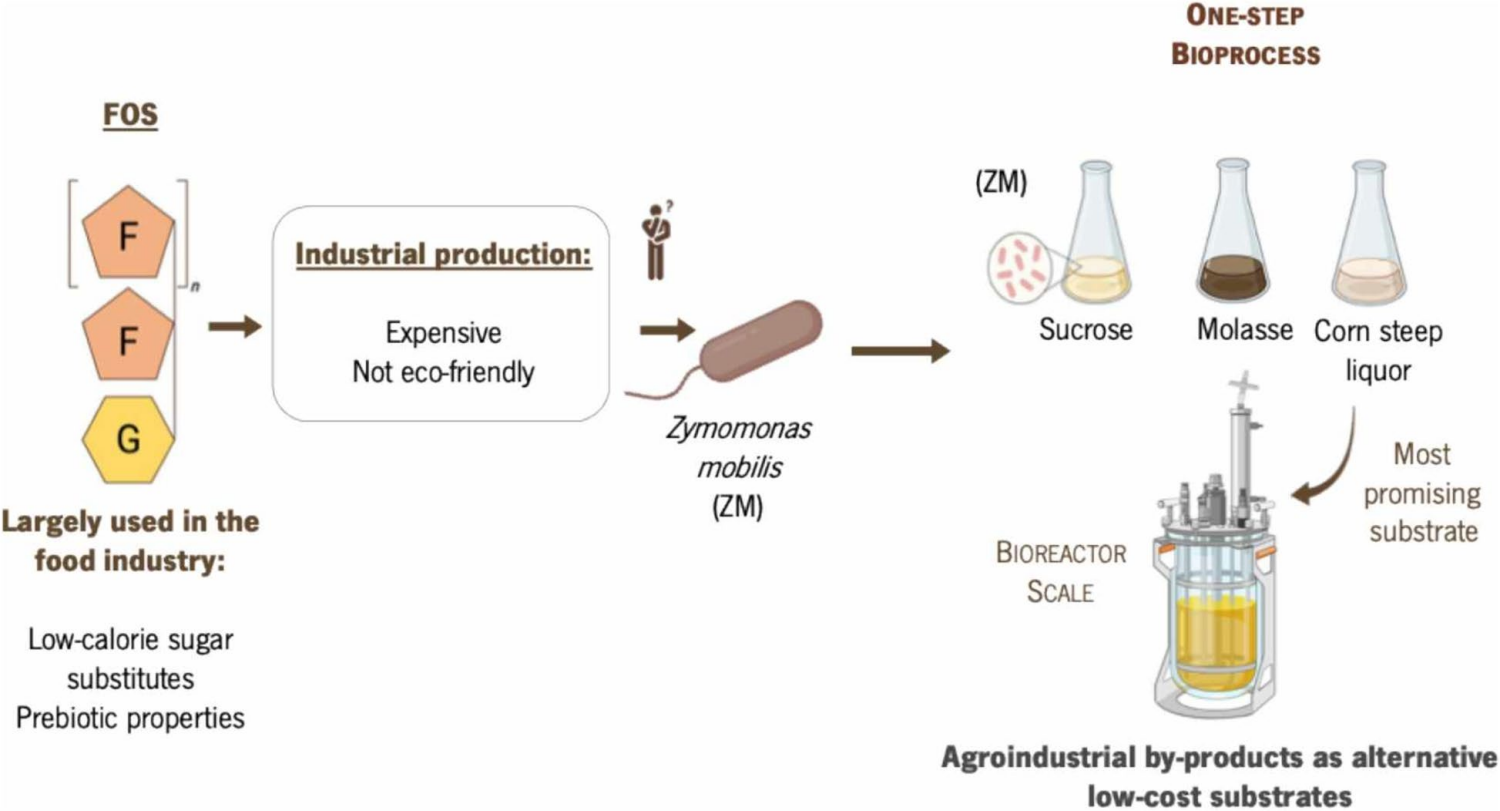

**Supplementary Information:**

The online version contains supplementary material available at 10.1186/s40643-025-00887-4.

## Introduction

The gut microbiota is vital for maintaining human health, influencing many physiological functions. As a result, there is a growing interest in strategies to modulate the microbiota for enhanced well-being. This has led to a shift in consumer behavior, with increasing focus on the health benefits of food beyond basic nutrition. In this context, prebiotics, a substrate that is selectively utilized by host microorganisms conferring a health benefit, have gained significant attention for their potential health benefits (Gibson et al. 2017).

Among the various types of prebiotics, fructooligosaccharides (FOS) are particularly important (Dominguez et al. 2014). FOS can be classified into different types according with the nature of linkage established between the monosaccharide residues, namely inulin-FOS (1F-FOS), levan-FOS (6F-FOS), neo-FOS (6G-FOS) and mixed levan (Flores-Maltos et al. 2016; Singh et al. 2017). Inulin-FOS are linear non-reducing chains of fructosyl units linked by β-(2 → 1) bonds (Hill et al. 2017). Usually, native inulin is processed and transformed into FOS or short chain fructans (scFOS), which will mainly derive in 1-kestose (GF2), nystose (GF3) and 1-β-fructofuranosyl-nystose (GF4) (Flores-Maltos et al. 2016). Levan-FOS are polysaccharides consisting of β-(2 → 6) linked fructosyl units (Hill et al. 2017). The levan molecule has a wide range of molecular weights that depend upon the source of the polysaccharide, being 6-kestose the smallest type of levan-FOS (Hill et al. 2017). The Neo-FOS are fructosyl units directly linked by β-(1 → 2) bonds to a fructofuranosyl residue of sucrose, which enables the elongation of the chain. One example of this compound is neokestose (Flores-Maltos et al. 2016). Mixed levans are a type of fructan composed of both β-(2 → 6) and β-(2 → 1)-linked fructofuranosyl units, which provide them with a hybrid structure combining features of levan- and inulin-type fructans. Bifurcose serves as the backbone for elongation into more complex mixed levans through enzymatic processes involving 1-FFT and 6-SFT enzymes (Vijn and Smeekens 1999). Industrially, FOS can be produced by microbial processes using two different strategies, namely one-step or two-step approaches. In the first approach, whole-cell microorganisms were used, either suspended or immobilized, as a biocatalyst enabling the production of the enzyme and FOS, simultaneously. As an alternative, FOS production in two-step bioprocess begins with the production of enzymes by microbial fermentation followed by the purification and incubation of the extracted enzyme(s) with sucrose to synthesize FOS (Tseng-Hsing Wang 2015; Braga et al. 2022a). However, one of the main bottlenecks in the production of FOS is the inhibition of these enzymes by glucose. As a result, at the end of the fermentation, a portion of sucrose remain unreacted (Sheu et al. 2013a).

Although FOS production has been widely reported by a variety of microorganisms, the use of *Zymomonas mobilis* strains is currently under-explored despite their great potential for producing these carbohydrates in one-step bioprocesses (Braga et al. 2021). This bacterium can survive in media with high glucose (up to 400 g L⁻^1^) and ethanol (up to 160 g L⁻^1^) concentrations. It has GRAS status, making it applicable in the food and pharmaceutical industries (Zhang et al. 2019). Additionally, it naturally produces valuable compounds such as levan, sorbitol, and FOS. Sucrose metabolism in *Z. mobilis* involves three enzymes, namely intracellular sucrase (*sacA* gene), extracellular levansucrase (*sacB* gene), and extracellular invertase/sucrase *(sacC* gene). The function of *sacA* remains unclear due to the unknown sucrose transport mechanism into the bacterium. The *sacC* gene encodes an enzyme that only hydrolyzes sucrose, responsible for about 70% of extracellular sucrase activity, with higher specific activity than levansucrase. The levansucrase enzyme (*sacB*) converts sucrose into levan and FOS (Goldman et al. 2008; Santos-Moriano et al. 2015; Braga et al. 2022b).

The growing need for sustainable and cost-effective biotechnological processes has led to an increased focus on utilizing alternative low-cost and renewable resources, such as agro-wastes, by-products and lignocellulose derived from forestry and agriculture residues. This is particularly relevant given the high cost of carbon and nitrogen sources in industrial media, which can account for 30–40% of production costs (de la Rosa et al. 2019). These by-products, rich in crude proteins and complex carbohydrates, can be valuable nutrients for microbial growth and enzyme synthesis (de la Rosa et al. 2019).

One promising approach involves using engineered strains *of Z. mobilis* to transform these renewable resources into valuable products (Braga et al. 2021). *Z. mobilis* can use various agro-industrial wastes, residues and by-products, such as sugarcane, sweet sorghum, carob, sugar beet, waste paper sludge, and sweet potatoes, to produce compounds like ethanol, levan, sorbitol, and gluconic acid (Braga et al. 2021).The production of FOS using industrial by-products has already been reported, with sugarcane by-products such as bagasse and molasses showing significant potential. Molasses, a by-product of the sugar industry, is particularly rich in fermentable sugars, vitamins, polyphenols, and minerals, and is produced in large quantities globally (de la Rosa et al. 2019; Šarić et al., 2016). Its heterogeneous composition, influenced by factors such as sugarcane variety and industrial processing, makes it an attractive substrate for fermentation processes, including the production of bioethanol, glutamate, acetone, and citric acid (Corrado et al. 2023; Kabeyi and Olanrewaju 2023). However, the molasses excess is often disposed in landfills, raising environmental concerns (Sen and Baidurah 2021). Given its nutrient-rich composition, molasses has been evaluated as an alternative carbon source for FOS production. Studies have shown that using molasses can result in higher FOS yields compared to pure sucrose (Khatun et al. 2021; Shin et al. 2004). Despite the known applications of *Z. mobilis* in producing various compounds from molasses, its potential for FOS production in such media has yet to be fully explored. Corn steep liquor (CSL), a by-product of the corn wet-milling process, also holds potential as a nitrogen source for FOS production due to its high protein content and rich nutrient profile (Amado et al. 2017; Loy and Lundy 2019). CSL has been used in fermentation processes to produce various compounds, but its application in FOS production remains underexplored (Zhou et al. 2022). Using CSL could enhance the sustainability and cost-effectiveness of the production process while reducing environmental pollution (Jiao et al. 2022).

The main goal of this work was to study the potential use of agro-industrial by-products as low-cost substrates in FOS production. Firstly, molasses and CSL were evaluated as alternative carbon and nitrogen sources for FOS production with *Z. mobilis*. By utilizing these cost-effective substrates, this study aims to demonstrate a novel approach for enhanced prebiotic production, addressing both economic and environmental considerations. This approach not only seeks to reduce production costs but also aims to mitigate environmental impact by repurposing industrial by-products. The findings herein gathered could pave the way towards more sustainable and economically viable production methods in the food and pharmaceutical industries.

## Materials and methods

### FOS production experiments

*Z. mobilis* ZM4 (ATCC 31821) was cultivated for 12–14 h in a 250 mL Erlenmeyer flask containing 50 mL of modified Rich Medium (RM) without pH control (20 g L^−1^ glucose, 10 g L^−1^ YE, 2 g L^−1^ potassium phosphate monobasic (KH_2_PO_4_), 1 g L^−1^ ammonium sulphate ((NH_4_)_2_SO_4_) and 2.04 g L^−1^ magnesium sulphate (MgSO_4_), with an initial pH of 5.5/6) at 30° C, without agitation (Hu et al. 2021).

In FOS production experiments different media were tested: molasses, CSL (Corn step liquor), and CSL and molasses (CSLM); sucrose concentrations were set according to the concentration of sugars present in molasses.

Sugarcane molasses (provided by RAR: *Refinarias de Açúcar Reunidas*, S.A.; Portugal) and CSL (provided by COPAM: *Companhia Portuguesa de Amidos*, S.A; Portugal) were used as alternative substrates for FOS production by *Z. mobilis* ZM4. Molasses contained 813.8 g L^−1^ sucrose, 30.9 g L^−1^ glucose and 3.6 g L^−1^ fructose, whereas CSL contained 75 g L^−1^ carbohydrates and 5 g L^−1^ protein (Gudiña et al. 2015).

### Experiments at shake flask scale

*Z. mobilis* ZM4 cells were pre-grown in RM medium as described in Sect.  FOS production experiments. *Z. mobilis* ZM4 cells were cultivated overnight, centrifuged for 10 min at 8000 rpm, and then resuspended in RM_Suc media (10 g L^−1^ Yeast extract, 1 g L^−1^ (NH_4_)_2_SO_4_, 2 g L^−1^ KH_2_PO_4_, 2.04 g L^−1^ MgSO_4_.7H_2_O (2.04 g L-1), 40 g L^−1^ sucrose). After 24 h, the inoculum was centrifuged (8000 rpm, 10 min) and used to inoculate 100 mL of production medium with molasse or/and CSL, in 250 mL flasks, with an optical density at 600 nm (OD_600nm_) of 0.20 and incubated at 30 °C under static conditions.

In the molasses experiments, the culture medium was formulated with different molasses concentrations, equivalent to 150, 200 and 350 g L^−1^ sucrose. Culture media were supplemented with yeast extract (YE), NaCl, MgSO_4_.7H_2_O, KH_2_PO_4_ and (NH_4_)_2_SO_4_ at the same levels as production medium. In the CSL experiments, two different combinations of CSL and YE concentrations were analysed, maintaining the final nitrogen source concentration at 20 g L^−1^: 10 g L^−1^ CSL + 10 g L^−1^ YE; 12 g L^−1^ CSL + 8 g L^−1^ YE. Culture media were supplemented with sucrose, NaCl, MgSO_4_.7H_2_O, KH_2_PO_4_ and (NH_4_)_2_SO_4_ at the same levels as the production medium. Molasses and CSL were further combined in the concentrations that increased the production of FOS. The culture media was supplemented with NaCl, MgSO_4_.7H_2_O, KH_2_PO_4_ and (NH_4_)_2_SO_4_ at the same levels as production medium. Samples were collected periodically to analyse the production of FOS, sorbitol, and ethanol, sucrose consumption, as well as the consumption/production of monosaccharides (glucose and fructose).

### Experiments at bioreactor scale

A pre-culture of *Z. mobilis* ZM4 in RM_Suc, prepared as described in the Sect. Experiments at shake flask scale, were used to inoculate the bioreactor with an OD_600nm_ of 0.2.

Experiments were performed using a 2-L DASGIP Parallel Bioreactor System (Eppendorf, Hamburg, Germany) with a working volume of 400 mL. The temperature was maintained at a set-point of 30 °C, and the experiments were carried out at 100 rpm, under microaerophilic conditions (without aeration) and without pH control (the initial pH of the medium ranged from 4.5 to 5.0). Before inoculation, sterile nitrogen was circulated through the medium for 1 h. A pre-culture, prepared as outlined in the Sect. Experiments at shake flask scale, was utilized to inoculate the bioreactor with an OD_600nm_ of 0.2.

The production medium previously optimized, was used for bioreactor batch studies. Samples were collected periodically to analyse the concentration of FOS, sorbitol, ethanol, sucrose, glucose and fructose.

### Analytical methods

The sorbitol and ethanol concentrations were assessed through high-performance liquid chromatography (HPLC) utilizing a JASCO system coupled with a refractive index (RI) detector (RI-2031) (Braga et al. 2022b). Analysis was conducted using an exchange column (Aminex HPX-87H, 300 × 7.8 mm, Bio-Rad), maintained at 60 °C. Samples were injected at a flow rate of 0.5 mL min^−1^, using H_2_SO_4_ (5 mM) as the eluent for 30 min.

For quantification of FOS (1-kestose, 6-kestose, nystose, neokestose, and 1-β-fructofuranosyl-nystose), fructose, glucose, and sucrose, samples were analysed by HPLC using a JASCO system with an RI detector, and an Asahipak NH2P-50 4E column (5 µm, 250 × 4.6 mm, Shodex), according with (Nobre et al. 2018). Samples were injected at a flow rate of 0.9 mL min^−1^, at 30 °C, using a mixture of acetonitrile in pure water (75:25% v/v) and 0.04% (v/v) ammonium hydroxide as the mobile phase.

### Statistical analysis

The results were presented as the mean values of two independent experiments ± standard derivation. Unpaired t-tests were performed to assess statistical significance when necessary, and Tukey tests were used for post hoc comparisons. For the statistical analysis of data, GraphPad Prism version 8.0.1 (GraphPad. Software. Inc.) was used. When p-value was < 0.05, significant differences were considered.

## Results and discussion

### FOS production with corn steep liquor

CSL is a liquid by-product generated by the corn wet milling industry. It is rich in vitamins, minerals, amino acids and proteins, being an important source of nitrogen for many biotechnological processes (Maddipati et al. 2011; Henkel et al. 2012).

In this work, two different combinations of CSL and YE were evaluated, to determine the optimal concentration of CSL for FOS production (Table 1).

**Table 1:** Concentration of fructooligosaccharides (FOS), productivity and yield, obtained after a 48 h incubation period with different concentrations of corn steep liquor (CSL) and yeast extract (YE) by the *Zymomonas mobilis* ZM4 strain. The culture media contained 350 g L^−1^ of sucrose. The values presented correspond to the average of three independent experiments ± standard derivation

CSL + YE Concentration (g L^−1^)	FOS concentration (g L^−1^)	Productivity (g L^−1^ h^−1^)	Yield (g_FOS_ g_sucrose_^−1^)
10 + 10	62.98 ± 0.44	1.312 ± 0.009	0.21 ± 0.02
12 + 8	60.00 ± 0.44	1.250 ± 0.009	0.20 ± 0.008

In medium with 10 g L^−1^ CSL, the maximum FOS production was obtained after 48 h with a total FOS concentration of 62.98 ± 0.44 g L^−1^, with a productivity of 1.312 ± 0.009 g L^−1^ h^−1^ and a yield of 0.21 ± 0.02 g_FOS_ g_sucrose_^−1^. When grown in medium with 12 g L^−1^ CSL, *Z. mobilis* was able to produce 60.00 ± 0.44 g L^−1^ FOS, with a productivity of 1.250 ± 0.009 g L^−1^ h^−1^ and a yield of 0.200 ± 0.008 g_FOS_ g_sucrose_^−1^, after 48 h (Table 1). In fact, the obtained results demonstrated that an increase in CSL concentration did not significantly affect the FOS concentration (*p* > 0.05). Comparing the FOS concentration herein obtained with data described in the literature for synthetic medium, it can be concluded that FOS production is similar in medium containing CSL. Braga et al. (2022a) reported the production of 51.56 g L^−1^ FOS (productivity of 1.1 g L^−1^ h^−1^; yield of 0.162 g_FOS_ g_sucrose_^−1^) from 20 g L^−1^ of YE and 350 g L^−1^ sucrose, in a one-step process with *Z. mobilis* ZM4. The production of FOS in CSL with other microorganisms has already been reported (Maiorano et al. 2008; Ning et al. 2012). However, those studies were performed using a two-step approach, where the enzyme is firstly synthesised via microbial fermentation and then purified and incubated with the desired substrate to produce FOS (Ning et al. 2012). In contrast, the present study is the first to report the production of FOS by *Z. mobilis* ZM4 in a one-step bioprocess using by-product and residues as substrate.

Considering the FOS production profile attained during these experiments (Figure S1C and S1D), for both concentrations of CSL, the FOS mixture composition included 1-kestose, 6-kestose and nystose, with 1-kestose synthesised in higher amounts (40.76 ± 0.08 g L^−1^). This FOS profile is like the ones reported by Braga et al. (2022a) using synthetic medium with 350 g L^−1^ sucrose and Bekers et al. (2002) with sucrose syrup using *Z. mobilis* levansucrase.

Considering the results herein obtained, it was found that CSL can be used as an alternative nitrogen source in FOS production with *Z. mobilis* ZM4, when supplemented with YE. Additionally, 12 g L^−1^ CSL + 8 g L^−1^ YE was selected as the most attractive nitrogen source combination for FOS production.

### FOS production with molasses

To reduce the process production costs and improve the efficiency and industrial interest of the process, molasses was used as an alternative substrate in the production of FOS by the *Z. mobilis* ZM4 strain. Different molasses concentrations were tested to establish the best condition for FOS production. This bacterium can withstand sugars concentration up to 400 g L^−1^ (Zhang et al. 2019). Additionally, FOS production in *Z. mobilis* was proven to be more efficient in synthetic medium containing 350 g L^−1^ sucrose (Braga et al. 2022a). For this reason, a concentration of sucrose in molasses equivalent to 350 g L^−1^ was firstly tested (Table 2).

**Table 2:** Concentration of fructooligosaccharides (FOS), productivity, yield and residual sucrose, obtained after a 96 h incubation period with different concentrations of molasses by the *Zymomonas mobilis* ZM4 strain

Molasses concentration (g L^−1^)	FOS concentration (g L^−1^)	Sucrose concentration (g L^−1^)	Productivity (g L^−1^ h^−1^)	Yield (g_FOS_ g_sucrose_^−1^)	Time (h)
350	40.77 ± 0.97	173.92 ± 7.26	0.42 ± 0.11	0.41 ± 0.13	96
200	58.67 ± 1.64	21.49 ± 5.62	1.22 ± 0.03	0.37 ± 0.12	48
150	5.71 ± 0.58	0	0.079 ± 0.008	0.05 ± 0.02	72

The values presented correspond to the average of three independent experiments ± standard derivation

Analysing the sucrose consumption (Table 2), it was possible to observe that at the end of the experiment, 173.92 ± 7.26 g L^−1^ of this disaccharide was still present in the fermentative broth. Normally, in synthetic medium with the same amount of sucrose, this compound would be consumed after 48 h. Nevertheless, *Z. mobilis* ZM4 was able to produce FOS directly from molasses. Herein, a maximum FOS concentration of 40.77 ± 0.97 g L^−1^, was achieved at the end of the experiment (96 h). However, the FOS concentration achieved in this experiment, is 1.27-times lower (*p* < 0.05) than the FOS titter obtained in the previous experiments with 12 g L^−1^ CSL. Moreover, Braga et al. (2022a) also reported a high a FOS concentration (51.56 ± 0.22 g L^−1^) in a previous study with *Z. mobilis* cultivated in a medium with 350 g L^−1^ of sucrose.

The decrease in FOS production may be related to the presence of monosaccharides (glucose and fructose) in molasses. The presence of these sugars inhibits the activity of levansucrase, expressed by the *sacB* gene. Khandekar et al. (2014) found that, when glucose is added to the medium, the FOS synthesis rate and the yield decreases. These results suggest that glucose binds to the invertase active site for transfructosylation activity, inhibiting its activity. Hence, the initial glucose concentration (Figure S2A, 28.65 ± 2.52 g L^−1^) present in molasses and the production of this monosaccharide during the experiment may have decreased the production of FOS (maximum concentration of 34.57 ± 7.11 g L^−1^ for fructose and 37.71 ± 0.82 g L^−1^ for glucose at 96 h and 72 h, respectively).

In fact, it was already reported that using high initial substrate concentrations lead to incomplete fermentations (Tano et al. 2000). Cazetta et al. (2005) reported that high concentrations of molasses (equivalent to 300 g L^−1^ carbohydrates) also contain significant levels of salts (sodium, potassium, calcium, phosphorus, chlorine and sulphur), which may raise osmotic pressure above tolerable levels reducing cell viability. Consequently, a decreased cell viability can result in a lower sucrose consumption.

Although the results suggest that molasses equivalent to 350 g L^−1^ sucrose can potentially be used as substrate for FOS production, a decrease in molasses concentration could be beneficial for the bioprocess. Bearing this in mind, experiments using a concentration of sucrose in molasses equivalent to 150 and 200 g L^−1^ were conducted (Table 2).

Considering the sucrose intake profile of the experiment with 150 g L^−1^ molasses, it was possible to observe that this compound was totally consumed after 24 h. On the other hand, in medium with 200 g L^−1^ molasses (Table 2), almost all sucrose was consumed, with only 21.49 ± 5.62 g L^−1^ remaining in the fermentative broth at the end of the experiment. With this molasses concentration, *Z. mobilis* was able to produce 77.60 ± 0.50 g L^−1^ of fructose and 137.61 ± 10.29 g L^−1^ of glucose after 48 h and 72 h, respectively (Figure S3B). In the previous experiment with 350 g L^−1^ of molasses, lower amounts of glucose and fructose were accumulated in the fermented broth, which reinforces the hypothesis that high molasses concentrations (above 300 g L^−1^) reduces *Z. mobilis* cell viability (Cazetta et al. 2005). Regarding FOS production, with 150 g L^−1^ sucrose in molasses, a maximum FOS concentration of 5.71 ± 0.58 g L^−1^ was obtained after 72 h, with a productivity of 0.079 ± 0.008 g L^−1^ h^−1^ and a yield of 0.05 ± 0.02 g_FOS_ g_sucrose_^−1^. However, in medium with 200 g L^−1^, *Z. mobilis* ZM4 was able to produce 58.67 ± 1.64 g L^−1^ of FOS with a productivity of 1.22 ± 0.03 g L^−1^ h^−1^ and a yield of 0.37 ± 0.12 g_FOS_ g_sucrose_^−1^, after 48 h. Increasing the molasses concentration from 150 g L^−1^ to 200 g L^−1^ allowed a 10.01-fold increase in FOS production (*p* < 0.05). This observation was consistent with a previous study by Braga et al. (2022a), which reported that enhancing sucrose concentration from 100 to 200 g L^−1^ led to an increase in FOS concentration from around 3 g L^−1^ to 34 g L^−1^. In fact, levansucrase exhibits both transfructosylation and hydrolytic activities, and the balance between these two modes is highly dependent on sucrose concentration. At low sucrose concentrations, the enzyme predominantly displays hydrolytic activity, leading to the cleavage of sucrose into glucose and fructose rather than polymerizing fructose into levan or short chain fructooligosaccharides. This is because the availability of suitable acceptor molecules (other sucrose or fructan molecules) is limited, which shifts the reaction toward hydrolysis rather than transfructosylation (Braga et al. 2022a, 2022b).

Comparing the results from these two conditions with the values provided in the literature for synthetic medium with 350 g L^−1^, 100 and 200 g L^−1^ of sucrose (51.56, 34 and 3 g L^−1^), 200 g L^−1^ molasses could be used as an alternative carbon source in order to maximize FOS production (Braga et al. 2022a), since a 1.14-fold increase in FOS production (*p* < 0.05) was observed in comparison to the titter obtained in synthetic medium.

**Fig. 1:**
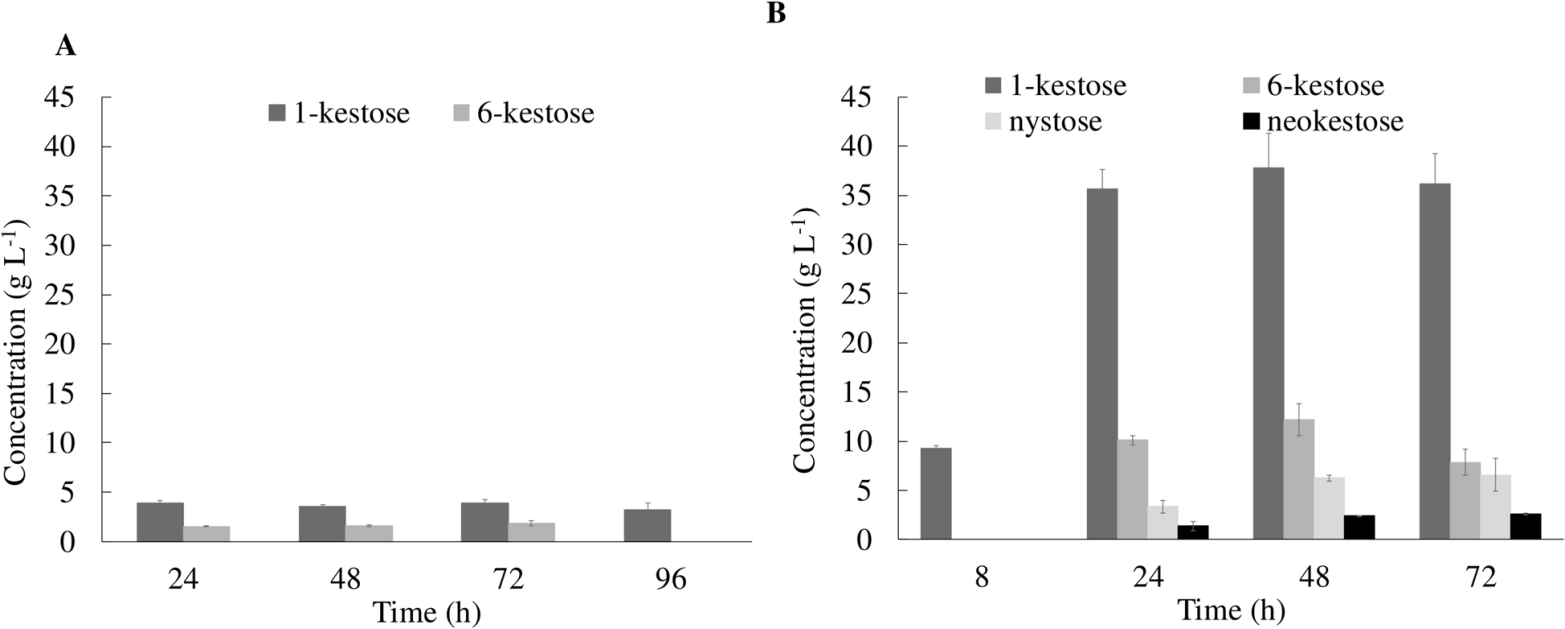
FOS production profile obtained in medium with 150 g L^−1^ of sucrose in molasse (**A**) 200 g L^−1^ of sucrose in molasse (**B**) using the *Zymomonas mobilis ZM4* strain. The values presented correspond to the average of two independent experiments ± standard derivation

The FOS production profile obtained is shown in Fig. 1. With 150 g L^−1^ molasses, only 1-kestose and 6-kestose are produced. However, with 200 g L^−1^, a more complex FOS mixture is obtained. This mixture includes 1-kestose, 6-kestose, nystose, and neokestose. Neokestose is a neoFOS that can be synthesized either from 1-kestose or neokestose (Santos-Moriano et al. 2015). The presence of neokestose in the mixture is beneficial to the prebiotic activity, since this trisaccharide has a superior bifidogenecity, as well as higher chemical and heat stability compared to the other FOS (Lee et al. 2015; Linde et al. 2012; Sheu et al. 2013b).

Among all the molasses concentrations tested (equivalent to 150, 200 and 350 g L^−1^), it was observed that FOS production by *Z. mobilis* can be achieved using molasses as an alternative carbon source. However, it was found that an equivalent sucrose concentration of 200 g L^−1^ is the best concentration for maximizing FOS concentration, and it was used in further optimization studies.

### FOS production in CSLM media

Considering the results previously obtained with CSL and molasses, FOS production by the wild-type strain was further evaluated in medium with both agro-industrial by-product. The best conditions of both experiments were used, resulting in a RM production medium with 200 g L^−1^ sucrose in molasses, 12 g L^−1^ CSL and 8 g L^−1^ YE.

**Fig. 2:**
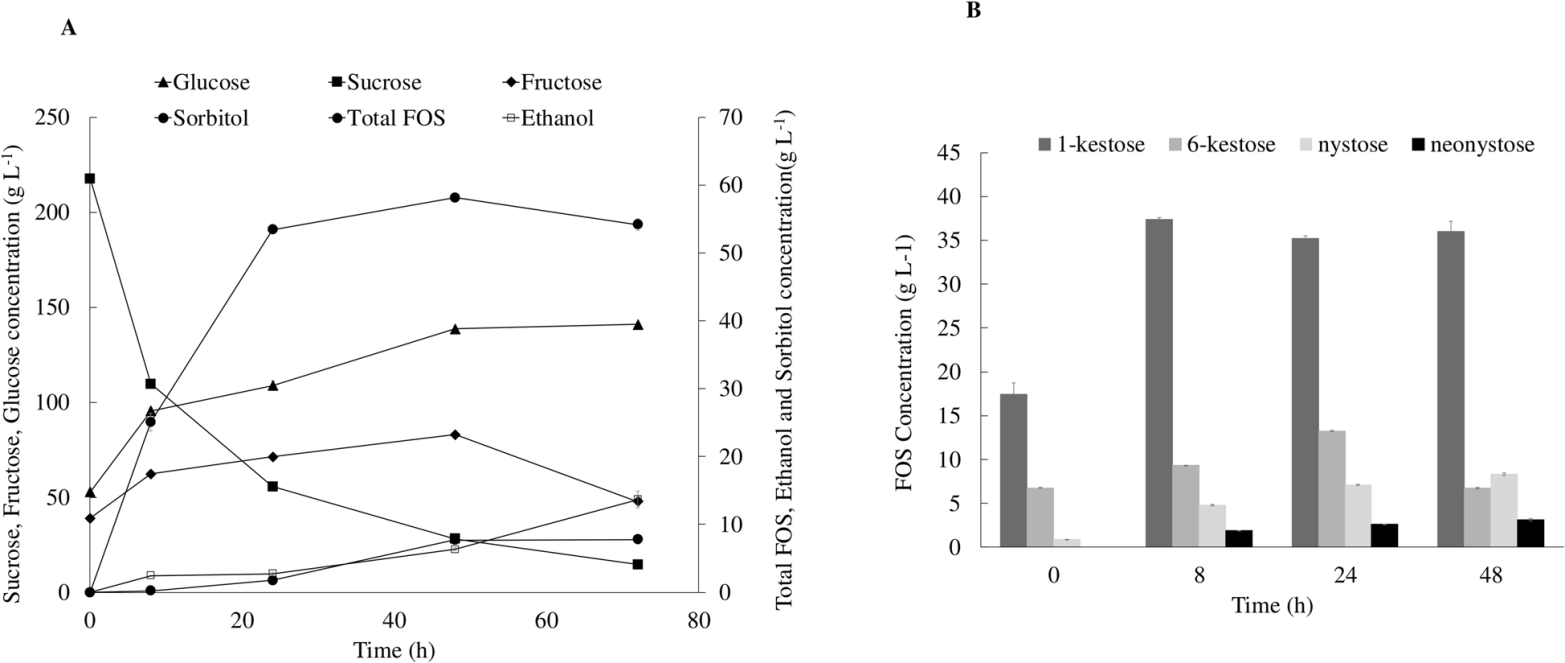
**A** Time course of fructooligosaccharides (FOS), ethanol, sorbitol, sucrose, fructose and glucose concentrations obtained in shake flask with 200 g L^−1^ of molasses, 12 g L^−1^ of corn steep liquor (CSL) and 8 g L^−1^ of yeast extract (YE). **B** FOS production profile. The values presented correspond to the average of two independent experiments ± standard derivation

Analysing the residual sugars profile (Fig. 2A), it was found that 14.55 ± 2.16 g L^−1^ sucrose was still present at the end of the experiment. Regarding the monosaccharide’s profile, a maximum concentration of 141.37 ± 0.66 g L^−1^ glucose and 82.19 ± 1.12 g L^−1^ fructose was obtained after 72 h and 48 h, respectively. These findings suggest that the high monosaccharides concentrations at the end of the experiment result from their initial concentrations in molasses. In media containing sucrose or a glucose-fructose mixture, *Z. mobilis* has been shown to produce sorbitol and ethanol (Barrow et al. 1984; Viikari and Berry 1988). At 72 h, 13.66 ± 1.23 g L^−1^ ethanol and 27.77 ± 2.33 g L^−1^ sorbitol were produced.

A maximum concentration of 58.15 ± 0.21 g L^−1^ FOS was synthesised after 48 h, with a productivity of 1.211 ± 0.004 g L^−1^ h^−1^ and a yield of 0.307 ± 0.003 g_FOS_ g_sucrose_^−1^. The FOS titter attained with CSLM media is similar to the values obtained in synthetic medium with 350 g L^−1^ sucrose by Braga et al. (2022a), as well as the ones previously obtained with 12 g L^−1^ CSL and 200 g L^−1^ sucrose in molasses. Additionally, Ning et al. (2012) reported the production of 238.12 g L^−1^ of neo-FOS (neokestose and neonystose) with *Xanthophyllomyces dendrorhous* in a medium with sucrose and CSL as carbon and nitrogen sources, respectively. Alvarado-Huallanco and Maugeri Filho (2011) also studied the synthesis of FOS using fructosyltransferase from *Rhodotorula sp.* in a medium containing molasses and 70 g L^−1^ of CSL, reporting a yield of around 57%. Despite the fact that the values reported in the literature are higher than the results obtained in this study, it is important to consider that those studies employed a two-step approach for FOS production, where the enzyme is firstly synthesised via microbial fermentation and then purified and incubated with the desired substrate to produce FOS (Braga et al. 2022a; Tseng-Hsing Wang 2015). When comparing the present study with other one-step FOS production processes, fungal-based systems often achieve higher titers and productivity. *Aspergillus oryzae* produced 119 ± 12 g L⁻^1^ of FOS from molasses and aguamiel in a single-step fermentation, achieving a yield of 0.64 ± 0.05 gFOS g⁻^1^sucrose and a productivity of approximately 4.96 g L⁻^1^ h⁻^1^ (de la Rosa et al. 2022). Nevertlhess, this work describes for the first time the production of FOS with the wild-type *Z. mobilis* ZM4 strain in an one-step approach with agro-waste residues.

The FOS production profile obtained in CSLM media exhibited four types of the prebiotic, namely 1-kestose, 6-kestose, nystose and neokestose, with 1-kestose being the FOS synthesised in higher amounts. At 48 h, 35.19 ± 0.35 g L^−1^ 1-kestose, 13.25 ± 0.05 g L^−1^ 6-kestose, 7.10 ± 0.06 g L^−1^ nystose and 2.60 ± 0.03 g L^−1^ neokestose were synthesised. 1-kestose titter tends to decrease after its maximum is achieved (at 24 h). Simultaneously, 6-kestose and nystose concentration tend to increase after this time point. This phenomenon can occur due to the conversion of 1-kestose into nystose, through transfructosylation reactions (Maiorano et al. 2008; Michel et al. 2016).

The results herein gathered show that CSL and molasses used as alternative nitrogen and carbon sources allowed the synthesis of FOS in a one-step approach with *Z. mobilis* ZM4, without any pre-treatment. This strategy contributes to a further optimization and decrease in the costs of the industrial production process.

### FOS production reaction

**Fig. 3:**
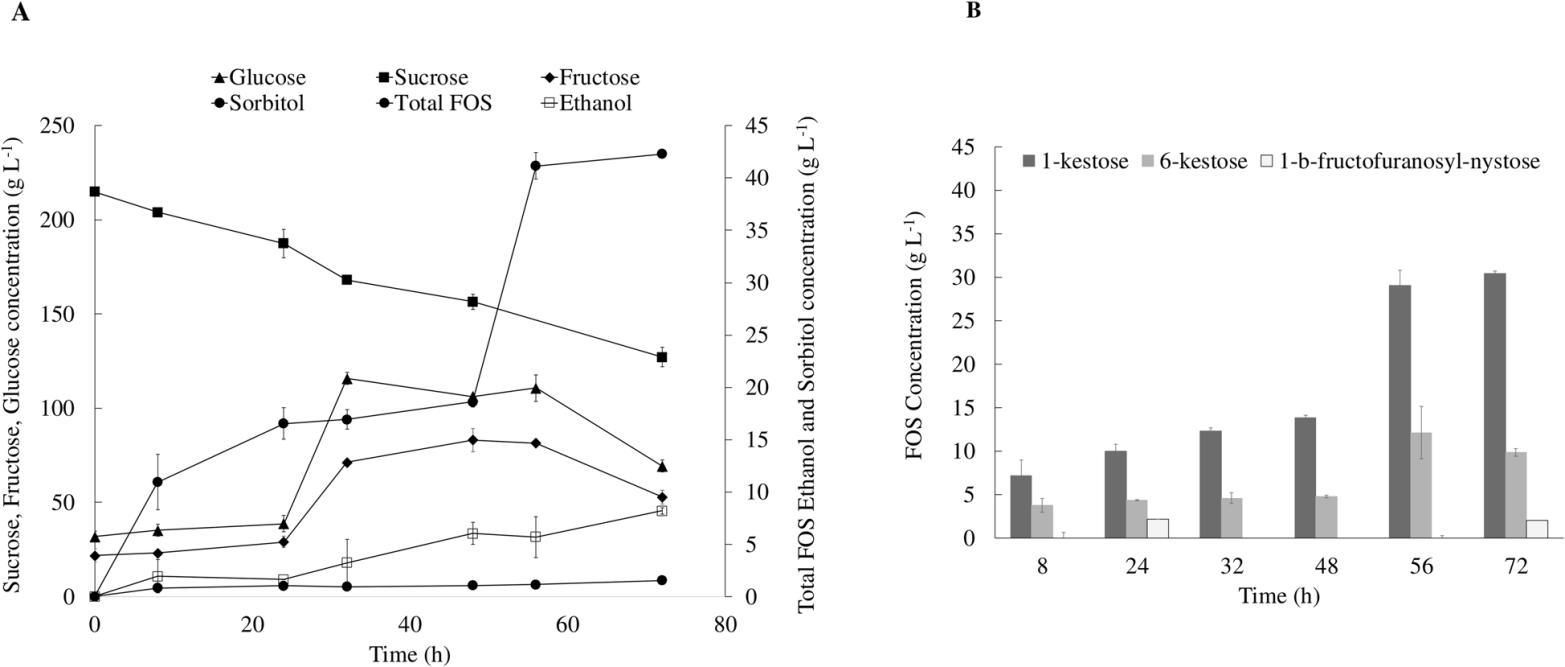
**A** Time course of fructooligosaccharides (FOS), ethanol, sorbitol, sucrose, fructose and glucose concentrations. **B** FOS production profile. This experiment was conducted in bioreactor under optimized conditions. The values presented correspond to the average of two independent experiments ± standard derivation

To further increase the FOS concentration, yield and productivity, experiments at a bioreactor scale were conducted using the medium composition previously optimized in the shake flask experiments (CSLM media: 200 g L^−1^ sucrose in molasses, 12 g L^−1^ CSL and 8 g L^−1^ YE). Figure 3 displays the sugars profile, as well as the production profiles for ethanol and sorbitol.

Sucrose concentration (Fig. 3A) decreased from 215.00 ± 0.97 g L^−1^ to 127.19 ± 4.42 g L^−1^ within 72 h. A sucrose consumption rate of 1.22 ± 0.22 g L^−1^ h^−1^ was achieved, which is lower compared to the shake flask results for CSLM medium (2.82 ± 0.45 g L^−1^ h^−1^). Letti et al. (2012) conducted a bioreactor study using *Z. mobilis* cultivated in soybean molasses, where around 50% of the initial sucrose present in the culture media was consumed. Within a 16-h period, the sucrose concentration declined from 116.8 g L^−1^ to 57.1 g L^−1^, indicating a sugars consumption rate of approximately 3.73 g L^−1^ h^−1^. The reduced sugar consumption observed, in this bioreactor experiment with CSLM, can be attributed to the different mechanical conditions between the shake flask and bioreactor, which affects the medium agitation.

A maximum FOS concentration of 42.31 ± 0.16 g L^−1^ was achieved at 72 h, yielding 0.482 ± 0.008 g_FOS_ g_sucrose_^−1^ with a productivity of 0.588 ± 0.002 g L^−1^ h^−1^. At this time point, the FOS mixture composition included 30.45 ± 0.25 g L^−1^ 1-kestose, 9.85 ± 0.41 g L^−1^ 6-kestose and 2.01 ± 0.32 g L^−1^ 1-β-fructofuranosyl-nystose (Fig. 3B).

The FOS concentration herein obtained is 1.37-times lower (*p* < 0.05) than those achieved in the shake flask experiments (Fig. 2B), where 58.15 ± 0.21 g L^−1^ FOS were synthesised. A similar behaviour was reported by de la Rosa et al. (2022) with *Aspergillus oryzae* in media with molasse and aguamiel. During the scale-up of the bioprocess, a decrease in FOS yields and concentrations was observed. In addition, the low FOS titter obtained in the bioreactor experiment with CSLM medium can also be associated with the high levels of monosaccharides (glucose and fructose) present in the fermented broth. Besides containing sucrose, molasses is a by-product rich in glucose and fructose (de la Rosa et al. 2019). Initially, the culture medium contained 21.82 ± 3.49 g L^−1^ fructose and 31.79 ± 2.90 g L^−1^ glucose. Due to sucrose hydrolysis, within 32 h, glucose titter reached 115.78 ± 3.06 g L^−1^, while fructose concentration peaked at 48 h with 83.12 ± 6.16 g L^−1^. In shake flask, after 48 h, glucose concentration reached 138.59 ± 1.18 g L^−1^. When glucose concentration exceeds 5.4 g L^−1^, it can exhibit an inhibitory effect on FOS synthesis (Lyness and Doelle 1983). Since glucose binds to the invertase active site for transfructosylation activity, an inhibition in its activity can be observed, resulting in an inferior FOS synthesis rate and yield (Khandekar et al. 2014).

Although the FOS titter achieved in CSLM media with *Z. mobilis* ZM4 is lower compared to the concentration reported by Braga et al. (2022a) in synthetic media (156.5 g L^−1^), similar yields were obtained (CSLM media—0.48 ± 0.04 g_FOS_ g_sucrose_^−1^; synthetic media—0.52 g_FOS_ g_sucrose_^−1^). For this reason, the yields obtained with *Z. mobilis* ZM4 in CSLM medium can be considered competitive comparing with the maximum yield usually found for FOS production with one-step processes (0.5–0.65 g_FOS_ g_sucrose_^−1^) (Dominguez et al. 2012; Nobre et al. 2018, 2019; de la Rosa et al. 2019; Braga et al. 2022a).

The implementation of CSLM media in this bioreactor experiment highlights its potential as an alternative media to produce FOS. However, the presence of reducing sugars in molasses is still the main drawback for FOS production with CSLM medium, and further optimization is required to enhance the concentration of FOS and ensure complete consumption of the sucrose present in the culture medium.

In fact, producing FOS using agro-industrial by-products like CSL and sugarcane molasses is a cost-efficient strategy compared to traditional synthetic media. These by-products, considered residues, dramatically reduce raw material costs. In contrast, synthetic media is expensive, contributing to a higher overall production cost. With this approach, it was possible to reduce the cost associated with the FOS production medium by approximately 5.5 times. Despite the potential need for more extensive purification processes due to impurities in agro-industrial by-products, the significant cost savings and environmental advantages, such as reduced waste disposal, render this method economically viable. Moreover, the growing demand for FOS, driven by their health benefits as prebiotics, underscores the scalability and profitability of this sustainable production method.

## Conclusions

The ability of *Z. mobilis* ZM4 to produce FOS by metabolizing sucrose presents a promising avenue for biotechnological applications. In this study, we demonstrated the potential of utilizing agro-industrial by-products, such as molasses and corn CSL, as alternative carbon and nitrogen sources, respectively, to enhance the economic viability of FOS production. Optimization of the culture medium composition revealed that 12 g L^− 1^ CSL and molasses equivalent to 200 g L^− 1^ sucrose resulted in the highest FOS production (58.15 ± 0.21 g L^−1^) with a yield of 0.307 ± 0.003 g_FOS_ g_sucrose_^−1^. Although scaling up the process to a bioreactor resulted in a lower FOS concentration (42.31 ± 0.16 g L^−1^), the yield remained promising, 0.482 ± 0.008 g_FOS_ g_sucrose_^−1^. These results underscore the potential of using CSL and molasses as sustainable and economical substrates for industrial FOS production. Although the titers obtained in the experiments at bioreactor scale were lower, CSL and molasses demonstrate significant potential as sustainable and cost-effective substrates for industrial FOS production. In fact, these residues offer a promising approach for the development of bioprocesses that not only decrease the production costs but also minimize environmental impact by repurposing agro-industrial by-products.

## Supplementary Information

Additional file1 (DOCX 2135 KB)

## Data Availability

The datasets generated during and/or analysed during the current study are available from the corresponding author on reasonable request.

## Abbreviations

CSL: Corn steep liquor
FOS: Fructooligosaccharides
YE: Yeast extract
CSLM: Corn steep liquor and molasses media
