# Supplementary material for: Agro-industrial by-products valorization for fructooligosaccharide production with Zymomonas mobilis
Source: Bioresour Bioprocess. 2025 Oct 7;12(1):110. doi: 10.1186/s40643-025-00887-4 (PMC12500493; doi:10.1186/s40643-025-00887-4)
Supplement: Supplementary file 1 — Additional file1 (DOCX 2135 KB) [file 40643_2025_887_MOESM1_ESM.docx]

**Agro-Industrial By-Products Valorization for Fructooligosaccharide Production with *Zymomonas mobilis***

Adelaide Braga^1,2^, Ana Benedita Maia^1^, Lígia R. Rodrigues ^1,2*^

^1^ CEB - Centre of Biological Engineering, Universidade do Minho, Campus de Gualtar, 4710-057 Braga, Portugal

^2^ LABBELS –Associate Laboratory, Braga, Guimarães, Portugal

* (Corresponding author)

E-mail: [lrmr@deb.uminho.pt](mailto:lrmr@deb.uminho.pt)

**Figure S1**

**
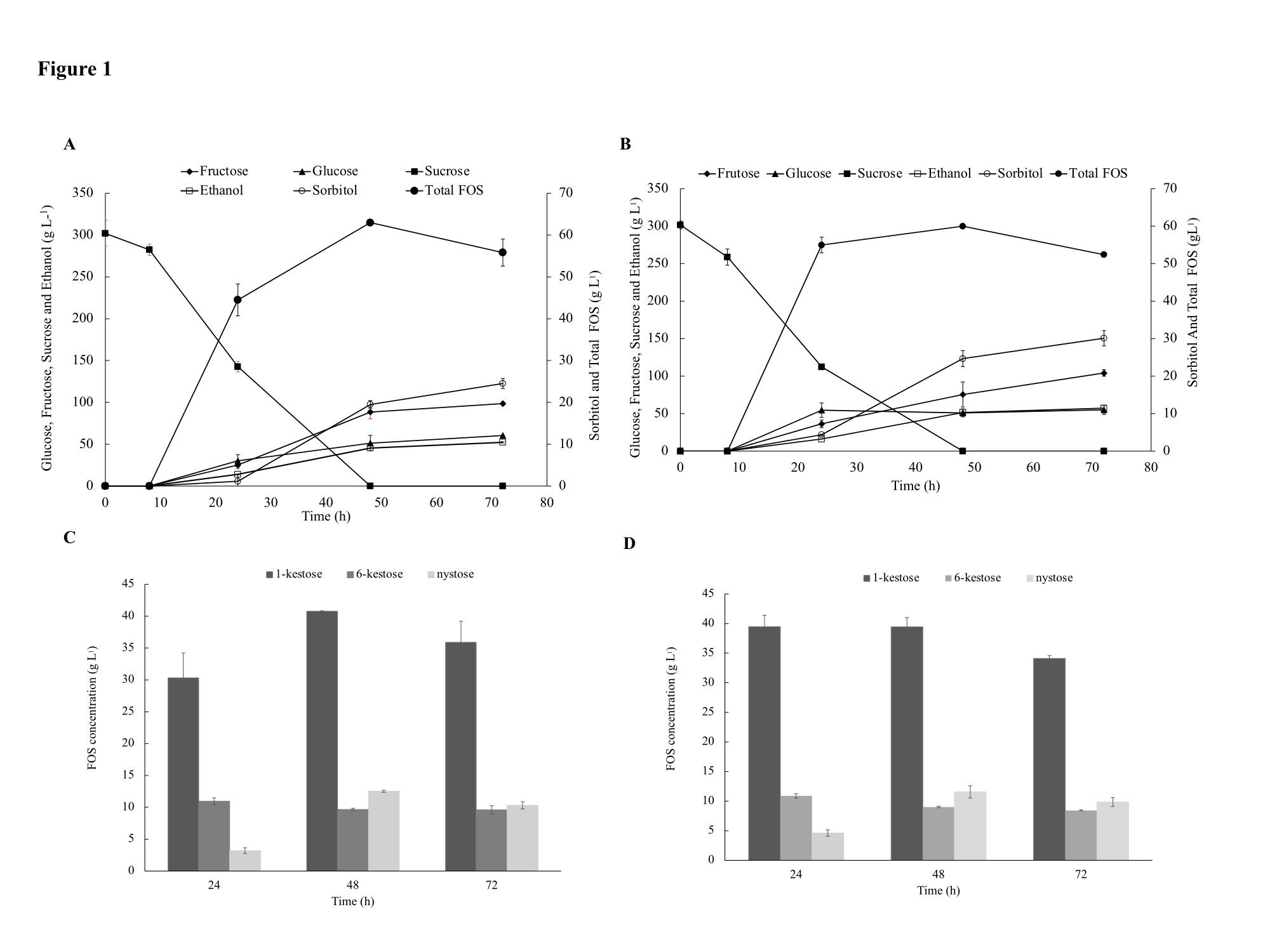
**

**Figure S1** - Time course of fructooligosaccharides (FOS), ethanol, sorbitol, sucrose, fructose and glucose concentrations obtained using the *Zymomonas mobilis* ZM4 strain with 10 g L^-1^ of CSL and 10 g L^-1^ of yeast extract (YE) (A) and 12 g L^-1^ of CSL and 8 g L^-1^ of YE (B) in shake flask. FOS production profile obtained in medium with 10 g L^-1^ of CSL and 10 g L^-1^ of YE (C) and 12 g L^-1^ of CSL and 8 g L^-1^ of YE (D). The values presented correspond to the average of three independent experiments ± standard derivation.

**Figure S2**

**
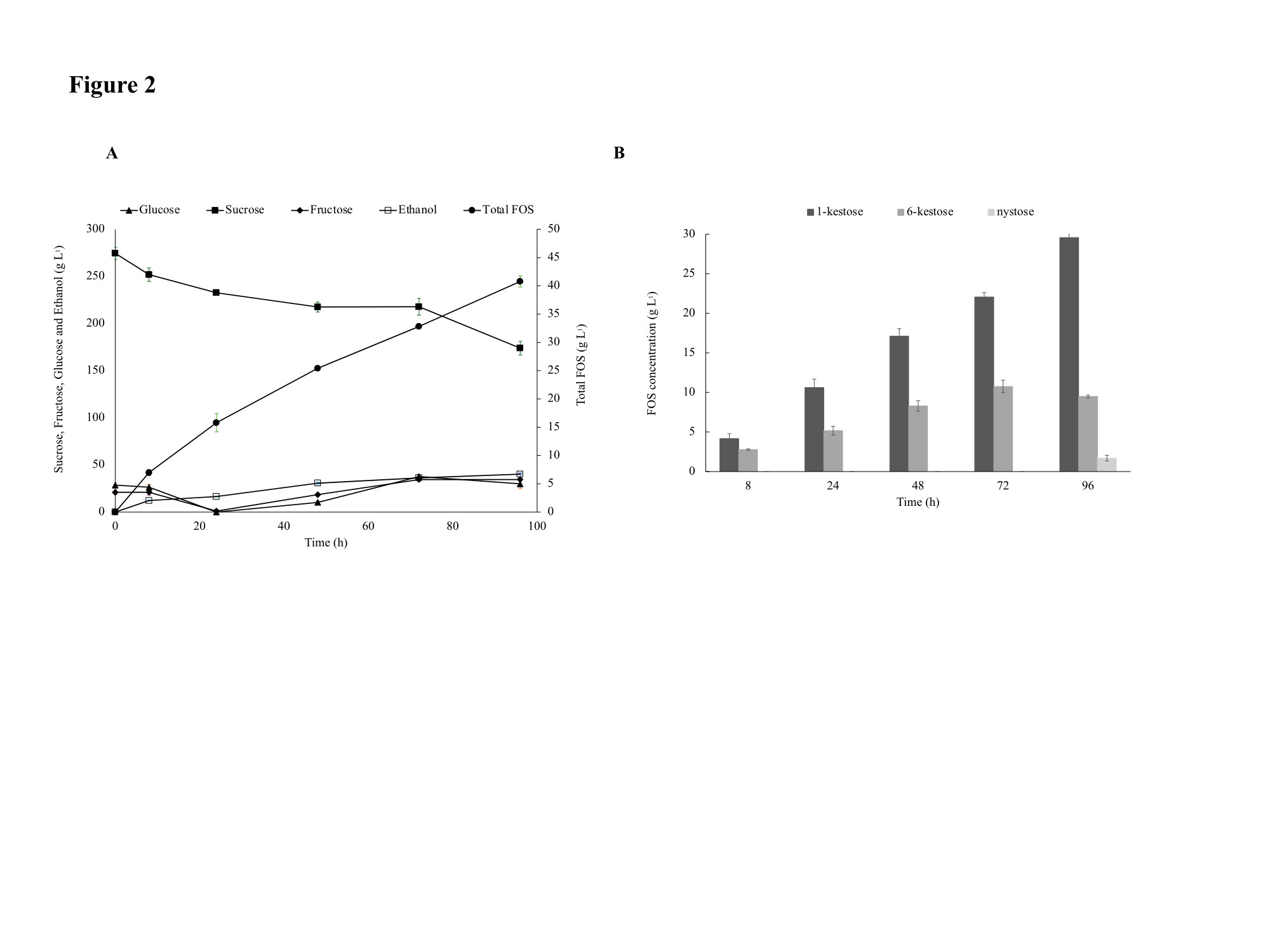
Figure S2 -** (A) Time course of fructooligosaccharides (FOS), ethanol, sorbitol, sucrose, fructose

and glucose concentrations, in shake flask with a molasse concentration equivalent to 350 g L^-1^

of sucrose. (B) FOS production profile. The values presented correspond to the average of two

independent experiments ± standard derivation.

**Figure S3**

**
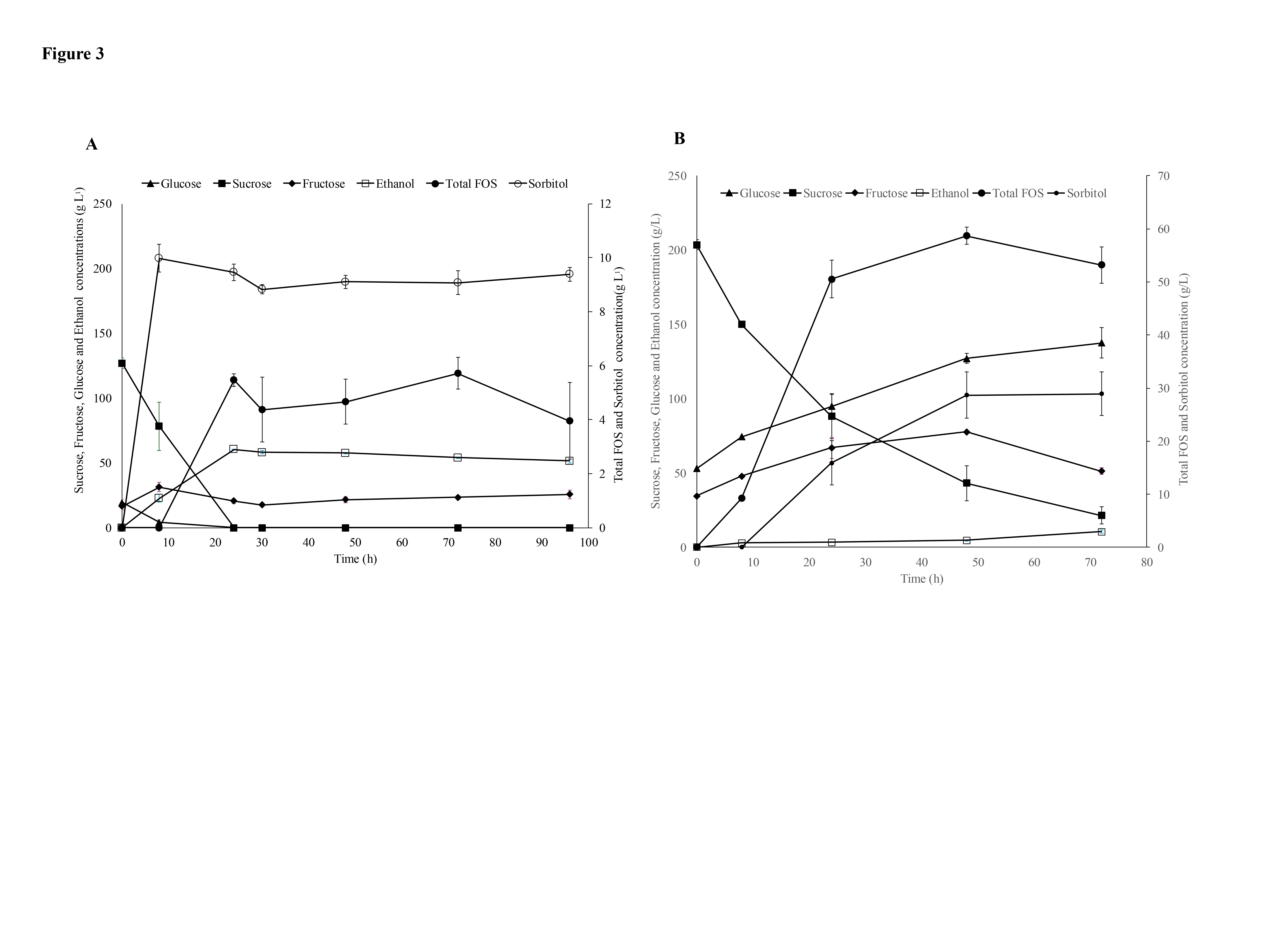
**

**Figure S3.** Time course of fructooligosaccharides (FOS), ethanol, sorbitol, sucrose, fructose and glucose concentrations obtained using the *Zymomonas mobilis ZM4* strain with 150 g L^-1^ of sucrose in molasse (A) 200 g L^-1^ of sucrose in molasse (B) in shake flask. The values presented correspond to the average of three independent experiments ± standard derivation.

**Figure S4**

**Figure S4.** Chromatogram profile of a sample showing retention times for fructose, glucose, sucrose, 1-kestose, 6-kestose, nystose and neokestose.
